# The incidence of increased ICP in ICU patients with non-traumatic coma as diagnosed by ONSD and CT: a prospective cohort study

**DOI:** 10.1186/s12871-016-0267-1

**Published:** 2016-10-25

**Authors:** Nawal Salahuddin, Alaa Mohamed, Nadia Alharbi, Hamad Ansari, Khaled J. Zaza, Qussay Marashly, Iqbal Hussain, Othman Solaiman, Torbjorn V. Wetterberg, Khalid Maghrabi

**Affiliations:** 1 Adult Critical Care Medicine, King Faisal Specialist Hospital & Research Centre, Riyadh, 11211 Saudi Arabia; 2 College of Medicine, Alfaisal University, P.O. Box 50927, Riyadh, 11533 Saudi Arabia; 3 Cleveland Clinic Abu Dhabi, P.O. Box 112412, Abu Dhabi, United Arab Emirates

**Keywords:** Optic nerve sheath diameter, Coma, Critical illness, Non-traumatic radiographic cerebral edema

## Abstract

**Background:**

Unexplained coma after critical illness can be multifactorial. We evaluated the diagnostic ability of bedside Optic Nerve Sheath Diameter [ONSD] as a screening test for non-traumatic radiographic cerebral edema.

**Methods:**

In a prospective study, mixed medical-surgical intensive care units [ICU] patients with non-traumatic coma [GCS < 9] underwent bedside ultrasonographic ONSD measurements. Non-traumatic radiographic cerebral edema [NTRCE] was defined as > 5 mm midline shift, cisternal, sulcal effacement, or hydrocephalus on CT.

**Results:**

NTRCE was identified in 31 of 102 patients [30.4 %]. The area under the ROC curve for detecting radiographic edema by ONSD was 0.785 [95 % CI 0.695–0.874, *p* <0.001]. ONSD diameter of 0.57 cm was found to be the best cutoff threshold with a sensitivity 84 % and specificity 71 %, AUC 0.785 [95 % CI 0.695–0.874, *p* <0.001]. Using ONSD as a bedside test increased the post-test odds ratio [OR] for NTRCE by 2.89 times [positive likelihood ratio], whereas post-test OR for NTRCE decreased markedly given a negative ONSD test [ONSD measurement less than 0.57 cm]; negative likelihood ratio 0.22.

**Conclusions:**

The use of ONSD as a bedside test in patients with non-traumatic coma has diagnostic value in identifying patients with non-traumatic radiographic cerebral edema.

## Background

Delayed elimination of sedatives and metabolic factors are considered as explanations for unexplained coma in intensive care unit [ICU] patients. Yet ‘new’ intracranial events may occur during the patient’s ICU stay, leading to unsuspected cerebral edema. Most patients with non-traumatic coma do not have direct intracranial pressure monitoring, either due to coagulopathy or because it is not routinely recommended. A reliable bedside screening test would be extremely useful to detect non-traumatic radiographic cerebral edema and thereby stratify patients that require urgent diagnostic and therapeutic interventions for delayed awakening. The optic nerve sheath diameter [ONSD] is now recognized as a sensitive and specific predictor of non-traumatic radiographic cerebral edema in patients with brain injury and is strongly correlated with both direct intracranial pressure measurements [1–6] and non-traumatic radiographic cerebral edema as diagnosed on brain imaging by CT scan [7–11] or MRI [12].

**Fig. 1:**
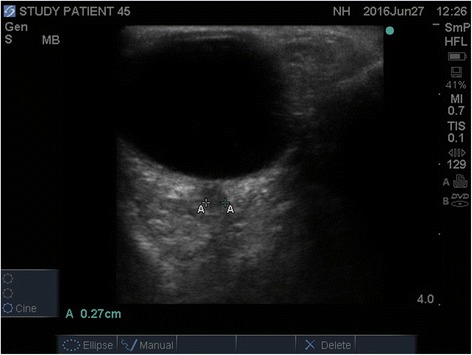
Optic nerve sheath diameter measurement by ultrasound

**Fig. 2:**
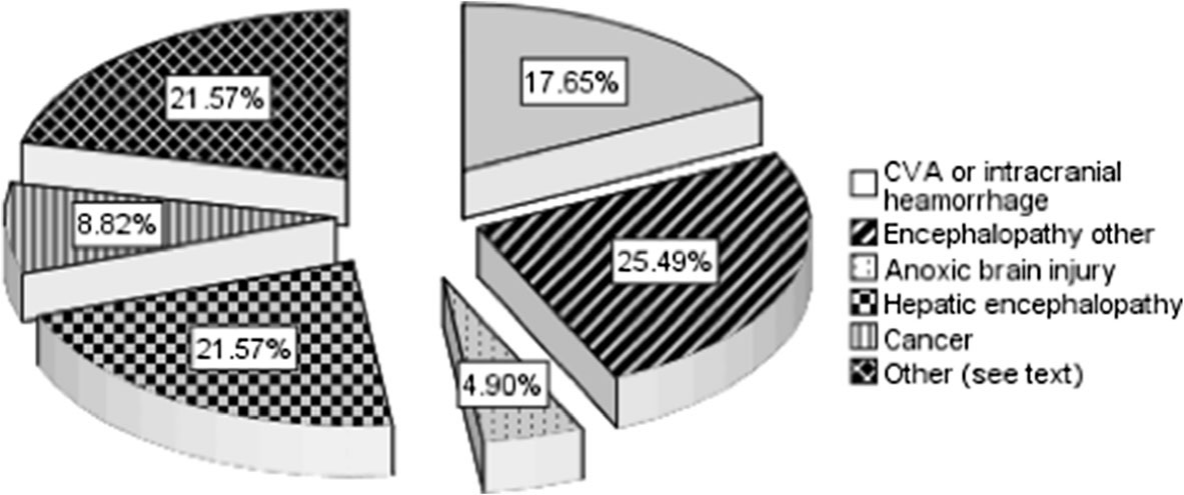
Final Cause of Coma

**Fig. 3:**
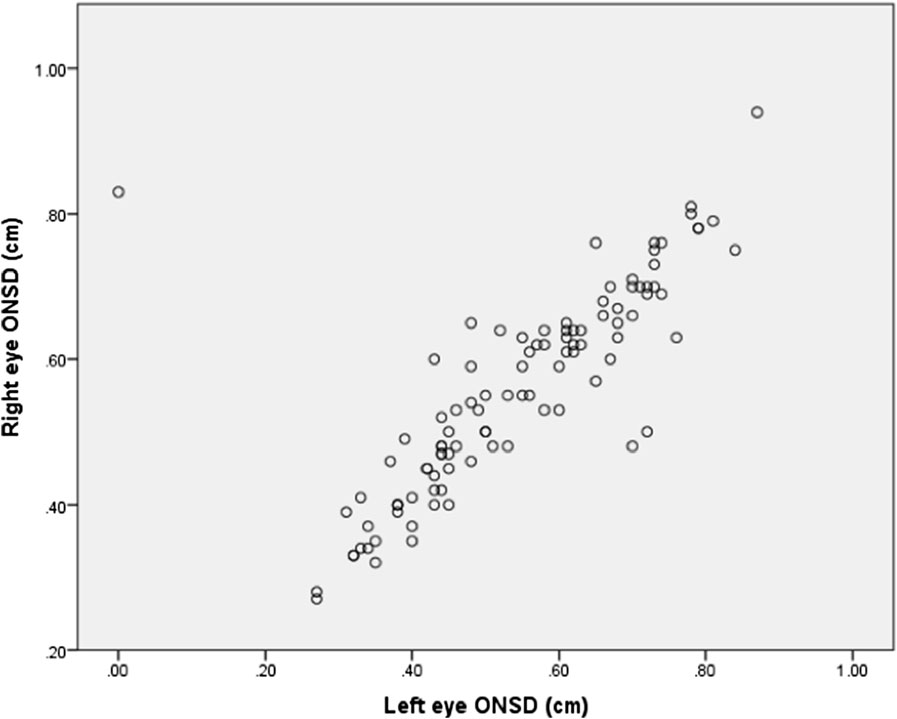
Bi-ocular correlations of optic nerve sheath diameter measurements; r2 value 0.86, *p* < 0.001

**Fig. 4:**
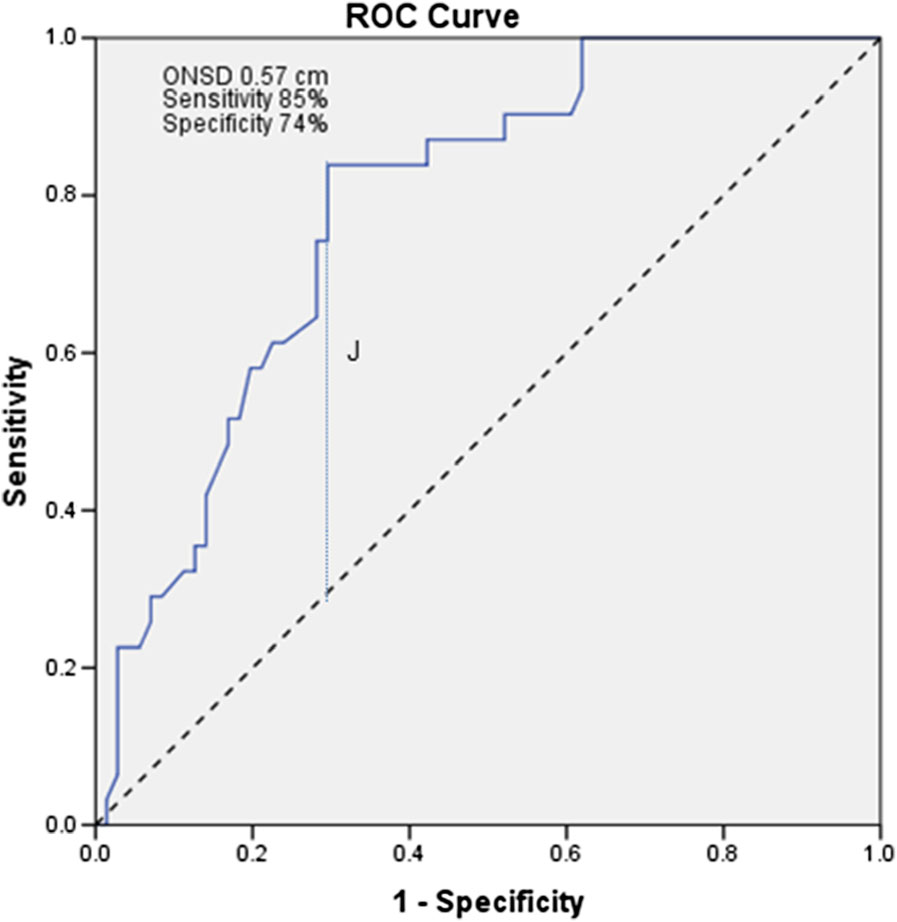
Receiver operating characteristics curve for discriminant optic nerve sheath diameter measurement for non-traumatic radiographic cerebral edema. AUC 0.785 [95 % CI 0.695–0.874, *p* <0.001]. Dashed line: Chance level; Vertical line [J] maximum value of Youden’s index for the ROC curve

The optic nerve attaches to the globe posteriorly and is wrapped in a sheath that contains cerebrospinal fluid. The optic nerve sheath is contiguous with the dura mater and has a trabeculated arachnoid space through which cerebrospinal fluid slowly percolates. Pressure increases around the brain are therefore transmitted to around the optic nerve and increase the diameter of the optic nerve sheath. In a study on brain injured patients in a neuro-ICU, an ONSD greater than 0.58 cm was shown to correlate significantly with an ICP > 20 cm H_2_0; *r* = 0.71, *p* < 0.001 [2]. So far, the evidence for ONSD has been restricted to patients with known or suspected brain injury, with very little data in general ICU patients.

The objective of this study was to assess the diagnostic utility of bedside ONSD measurements in critically ill patients previously considered not known to have an intracranial event and with unexplained coma. A secondary objective was to determine a cutoff ONSD value. Since ICU patients with non-traumatic coma usually do not have direct ICP measurements, we opted to use CT scan findings of cerebral edema [NTRCE] as the gold standard in this study.

## Methods

This was a prospective cohort study carried out on adult, critically ill patients admitted to mixed medical-surgical ICUs of a tertiary care, referral academic center. The study duration was from September 2014 to September 2015. Patients in whom the critical illness had resolved (defined as: stabilization or normalization of vital signs, no vasopressor or inotropic requirement and ventilator dependence only due to depressed mentation and described as ‘physiological improvement’) AND continued to have a unexplained non-traumatic coma [GCS < 9] despite no sedatives/ hypnotics for more than 48 h AND the treating ICU team considered further investigation of the comatose condition necessary, these patients met our inclusion criteria and were enrolled in the study. Exclusion criteria were known neurological conditions, ocular trauma, conjunctival edema or orbital edema. After verbal consent was obtained, all patients underwent ultrasonic measurement of ONSD in addition to conventional brain computerized axial tomographic scanning (CT). We included patients in whom a brain CT had already been ordered by the managing ICU team for evaluation of the comatose state. CT images were reviewed with the consultant radiologist for any obvious optic nerve abnormalities. The CT scans (which included the orbits) on any of the study patients did not reveal any local causes of optic nerve swelling. Patients who were admitted with decompensated liver cirrhosis, were managed accordingly, however when they continued to remain comatose (GCS <9) AFTER resolution of the inciting factor {GI bleed or improvement/ clearance of sepsis (resolution of sepsis criteria, improving procalcitonin, discontinued vasopressor support and negative cultures)} AND had not been on sedatives/ hypnotics AND the treating ICU team considered that a brain CT was warranted, they were enrolled in the study and ONSD measurements made. Concurrently serum ammonia measurements on venous blood were obtained (as per routine ICU protocol). We hypothesized that a relationship may exist between serum ammonia levels and the ONSD in patients with cirrhosis. Only concurrent ammonia and ONSD measurements were included and only a single measurement of the serum ammonia was recorded.

Non-traumatic radiographic cerebral edema [NTRCE] was defined by findings of midline shift [>5 mm], bilateral cisternal or sulcal effacement, or hydrocephalus [7, 8, 10, 11, 13] and confirmed by a consultant radiologist who was blinded to both clinical and ONSD measures. Demographic identifiers were collected; age, gender, comorbidity, admitting diagnosis, APACHE II scores, SOFA scores, neuroimaging and ICU outcomes. CT scans were obtained on ALL included patients, however a priori cutoff ONSD of 0.6 cm was recognized to be abnormal from brain trauma studies [2], therefore as a safety measure, the primary ICU team was notified if the diameter reached that value. Further management (testing, medical or surgical treatment) was left to the discretion of the primary team.

For the optic nerve sheath diameter, the ultrasound operators were two study investigators (AM, NH), trained by the master trainer (IH), and blinded to the clinical details of the patient. Each operator independently measured each optic nerve in real time and the measurements were confirmed by the master trainer off-line. Interrater variability was checked and is reported.

This manuscript follows the STROBE checklist for reporting observational studies.

### Technique of ONSD ultrasound measurement [14]

The diameter of the optic nerve sheath was measured using a *SonoSite M*-*Turbo*® system with a high resolution 7.5–10 MHz linear array ultrasound transducer probe (HFL38x transducer). Depth was adjusted so that the image of the eye filled the screen. Gain was adjusted to achieve acceptable imaging. Two measurements were taken for each optic nerve using the digital cursor and measurement software of the ultrasound machine. A large amount of standard water-soluble ultrasound transmission gel was applied to the patient’s closed eyelid. The globe was scanned in the transverse plane. The ONSD was measured at a predefined point 3 mm posterior to the globe in both eyes. ‘We measured ONSD once in each patient; two separate operators carried out each measurement within 30 min of each other’ (see Fig. 1). 

### Statistical methods

Consecutive patients meeting the inclusion criteria were enrolled. The primary outcome was the diagnostic ability of the ONSD to diagnose intracranial hypertension. In this proof of concept study, the projected sample size was 100 patients, using a sensitivity plot for pα 0.05 and estimating a prevalence of 0.5, sensitivity 0.9 and confidence interval 0.07. Sample size was calculated using nomograms designed for diagnostic studies, where a horizontal line is drawn from the estimated prevalence to the required confidence interval. Another line is drawn from that intersection vertically until it meets the expected sensitivity or specificity. A horizontal line is then drawn from that intersection to the right hand axis where the number of patients required may be estimated [15]. An interim analysis was carried out after 20 patients. Correlations between variables were checked using either Spearman or Pearson correlation coefficients, as appropriate. Receiver operator characteristic [ROC] curves were built to obtain maximal sensitivity and specificity of ONSD to non-traumatic radiographic cerebral edema. Youden index was calculated to identify the best ONSD cutoff threshold that discriminated between presence and absence of non-traumatic radiographic cerebral edema. For the evaluation of the diagnostic accuracy of the ONSD, we calculated the positive likelihood ratio [LR+] and negative likelihood ratio [LR-], where LR+ is the sensitivity/[1-specificity] and LR- is [1-sensitivity]/specificity. The intraclass correlation coefficient was obtained to assess inter-rater agreement. All analysis was performed and graphs were generated using SPSS [IBM SPSS version 22.0, Chicago, IL] software. Two-sided *p* values ≤ 0.05 determined statistical significance.

### Ethics, consent to participate and publish, and permissions

The study protocol was approved by the King Faisal Specialist Hospital and Research Center, Office of Research Affairs, Research Advisory Committee [RAC Proposal No. 2141 103]. The study was performed in accordance with the ethical standards laid down in the 1964 Declaration of Helsinki and its later amendments. Since no individual patient data/ images was presented, the Research Advisory Committee, Office of Research Affairs waived written consent. Verbal Informed consent to participate in the study and for consent to publish was obtained from the attendant next of kin or legally authorised representative and documented in the patient’s medical record, as approved by the ethical review committee. CT scans were obtained on ALL included patients, however a priori a cutoff ONSD of 0.6 cm was recognized to be abnormal from brain trauma studies [2], therefore as a safety measure, the primary ICU team was notified if the diameter reached that value. If the ONSD was found to be increased [> 0.6 cm] which may suggest non-traumatic radiographic cerebral edema, the primary ICU team was informed of the possibility of intracranial hypertension and an urgent brain imaging study recommended. It was also recommended that medical therapy (hyperosmolar saline or mannitol) be considered but the final management was left to the discretion of the treating ICU physicians. Once the CT scan was obtained, the primary ICU team instituted either medical or surgical therapy as indicated in case of cerebral edema.

## Results

From September 2014 to September 2015, we enrolled 102 patients; 42 female [41 %], mean age 58 ± 20 years, mean APACHE II score 24.2 ± 5.3, mean SOFA score 10.8 ± 2.1.

Non-traumatic radiographic cerebral edema was confirmed by CT scanning in in 30 % [31 patients]. Final cause of coma was identified as septic or metabolic encephalopathy in 25.4 % [26 patients], new intracranial vascular event in 17.6 % [18 patients], anoxic brain injury 4.9 % [5 patients], hepatic encephalopathy 21.5 % [22 patients], intracranial malignancy 8.8 % [9 patients] and others [intracranial infection, reversible posterior leukoencephalopathy syndrome (RPLS), subclinical seizures] in 21.5 % [22 patients] (see Fig. 2).

ONSD measurements correlated highly between both eyes, Spearman’s rho = 0.86, *p* ≤ 0.001. Consecutive ONSD measurements by two observers showed substantial agreement with a correlation coefficient of 0.80 [95 % CI 0.51–0.92] for the left eyes and 0.85 [95 % CI 0.61–0.94] for the right eyes (see Fig. 3).

ROC curves were constructed to establish the sensitivity and specificity of ONSD to predict NTRCE. An ONSD diameter of 0.57 cm. predicted NTRCE with sensitivity 84 % and specificity 71 %, AUC 0.785 [95 % CI 0.695–0.874, *p* < 0.001] (see Fig. 4). Using ONSD as a bedside test increased the post-test odds ratio [OR] for NTRCE by 2.89 times [positive likelihood ratio], whereas given a negative ONSD test [ONSD measurement less than 0.57 cm] the post-test odds ratio for NTRCE also decreased markedly [negative likelihood ratio 0.22].

Serum ammonia levels were obtained from patients with liver cirrhosis; mean serum ammonia was 78.7 (range 22–213). A strong correlation was observed between ammonia levels and ONSD, Spearman’s rho 0.73, *p* 0.025.

## Discussion

In this prospective study on a mixed population of ICU patients with non-traumatic coma, bedside ONSD measurements proved a reliable diagnostic tool to screen for non-traumatic radiographic cerebral edema. A cutoff ONSD of 0.57 cm appears to reliably predict NTRCE in more than 80 % of patients.

Optic nerve sheath diameter measurement is a relatively recent application of bedside ultrasound and has been described to correlate highly with direct measurement of ICP. In 37 patients in a neuro-ICU, Geeraerts [2] reported significant relationships between ONSD and ICP [*r* = 0.71, *p* < 0.0001] with changes in ICP strongly correlating with changes in ONSD [*r* = 0.73, *p* < 0.0001]. Moretti [4] in 63 patients with intracranial hemorrhage reported an ONSD-ICP correlation coefficient of 0.70 [95 % CI 0.58–0.79] and an optimal ONSD cut-off point of 0.52 cm to predict raised ICP [> 20 mmHg] with 93.1 % sensitivity [95 % CI: 77.2–99 %] and 73.85 % specificity [95 % CI: 61.5–84 %]. Similarly, Rajajee [5] in 65 patients with traumatic brain injury, intracranial hemorrhage, ischemic stroke and cancer reported the optimal ONSD for detection of ICP > 20 mmHg was >/=0.48 cm with a sensitivity of 96 % [95 % CI 91–99 %], specificity of 94 % [92–96 %]. The reliability of ONSD measurements in comparison with direct ICP measurements has been confirmed from diverse ethnic groups around the world; such as 98 Ugandan patients [16] with HIV and crypotococcal meningitis where the ONSD correlated with opening pressures on lumbar puncture [RR 2.39, *p* 0.003], 101 Chinese patients [17] with ONSD cutoff of 0.41 [sensitivity 95 %, specificity 92 %], 50 Iranian patients [18], *r* value 0.88, *p* < 0.05, 60 Indian patients [12] with meningitis and a cutoff ONSD 0.51 [sensitivity 84 %, specificity 100 %] and 25 with Tuberculous meningitis [ONSD 0.58, *p* < 0.001] [19]. ONSD measurements have also been useful for prognostication; in a retrospective study of 17 cardiac arrest patients [20], an ONSD > 0.54 cm predicted a poor neurologic outcome [positive likelihood ratio 3.1, negative likelihood ratio 0.23] and in 220 brain trauma patients, each 1 mm increase in ONSD was associated with a twofold increase in hospital mortality [OR 2.0, 95 % CI 1.2–3.2, *P* = 0.007] [21]. A recent metanalysis that pooled 478 patients concluded that ONSD had a sensitivity of 95 %, specificity 92 % with negative and positive likelihood ratios of 0.05 and 12.5 for a diagnosing non-traumatic radiographic cerebral edema when compared to brain CT [22]. ONSD measurement is proven to compare well with CT scan for the diagnosis of intracranial hypertension. However, previous studies were designed simply to confirm the association with intracranial hypertension, and/or were carried out primarily in patients with brain trauma, mountain sickness, cardiac arrest or in the emergency room/ triage evaluation [12, 19–53]. What has not been confirmed is whether the ONSD measurement performs well in comatose ICU patients and what is the optimal cut-off value to diagnose cerebral edema in this population. Our study did not attempt to simply repeat an association with intracranial hypertension, but instead to demonstrate, in the light of this known association, the *predictive ability of bedside optic nerve sheath diameter measurement in ICU patients who were comatose, with no history of trauma, and in whom ruling out cerebral edema was an important consideration,* a diverse population in whom the ONSD has not been widely described. We attempted to identify a *discriminant cutoff ONSD value* and present the reader with positive and negative likelihood ratios to improve post-test probability for cerebral edema. We obtained ONSD in patients who initially had a critical illness (as given in Table 1) and then had stabilization or normalization of vital signs, no vasopressor or inotropic requirement and ventilator dependence only due to depressed mentation. We used the term ‘physiological improvement’ to describe these patients. If these patients now continued to have a GCS <9 and the treating ICU team considered further investigation of the comatose condition necessary, these patients met our inclusion criteria and were enrolled in the study. We focused primarily on patients where invasive ICP monitoring is not done, either due to co-existing coagulopathy or not recommended. Therefore no direct ICP measurements were available. However as non-traumatic radiographic cerebral edema in this patient cohort is usually diagnosed by CNS imaging, and as confirmed to be a reliable correlate of direct ICP measurement by previous investigators [7, 8, 10, 11, 13], we used CT scan findings as a surrogate measure [NTRCE]. Though this may raise concerns regarding the accuracy of the diagnosis, this probably reflects standard practice for most medical ICUs in the ‘real world’, and we think that the reliance on CT imaging actually makes the results more generalizable. However we recognize this as a limitation in our study.

**Table 1:** Patient characteristics

Initial Cause of ICU Admission	*n* (%)
Severe Sepsis or Shock	35 (34.3 %)
Respiratory failure	22 (21.6 %)
Postoperative care	20 (19.6 %)
GI bleed	6 (6 %)
Congestive Heart Failure	7 (6.9)
Venous thromboembolism	3 (2.9)
Neuromuscular weakness	9 (8.7 %)

Since the CT images (which included the orbits) on any of the study patients did not reveal any local causes of optic nerve swelling and therefore the ONSD were considered representative of NTRCE. We also found a strong correlation between serum ammonia levels (venous) and the ONSD in patients with decompensated chronic liver failure. Whether this represents a direct relationship between hepatic encephalopathy and ammonia will need to be determined in larger studies.

Limitations of our study are that we did not specifically obtaining CT scans of the orbit, however we considered the CT brain with images of the orbit, as a sufficient screen for any obvious orbital nerve abnormalities. All CT scans were discussed with the reporting radiologist and no optic nerve abnormalities were detected. Furthermore, as our study was designed only to evaluate the ONSD as a reliable screening test in a patient with global CNS suppression in the ICU, we did not do a detailed neurological exam or record any specific localizing neurologic signs. Similarly, though we included all patients with a GCS < 9, we did not record individual GCS values and attempt to identify a relationship with the ONSD measurement. We also did not measure the effect of treatment on the ONSD. These are interesting relationships that could be explored by future studies.

In our patient cohort we found a very close correlation between bilateral ONSD measurements; Spearman’s rho = 0.86, *p* ≤ 0.001, however this raises an interesting point about possible explanations in case of a large difference between bilateral ONSDs and what exactly would constitute a ‘significant’ difference. A limitation of our study is that we did not conduct angiographic or vascular doppler assessments to explore differences in vascular drainage which may be important in case of differences between bilateral ONSD measurements.

This study therefore allows us to demonstrate the value of bedside ONSD measurement as a screening test and its application beyond previously described populations. We consider that our results will increase the confidence of bedside clinicians when dealing with similar patients, especially patients for whom transfer to CT maybe hazardous.

Measurement of the ONSD has been reported to be simple, easily learned, and reproducible. In 2002, Ballantyne [14] reported ± 0.01 intra-observer and ± 0.2–0.3 inter-observer variabilities. Bauerle in 2013 [54] described a significant correlation between ONSD measured by ultrasound and that measured on MRI [*r* 0.72, *p* < 0.001]. Hassen [55], in a retrospective study on 61 patients reported that ONSDs measured by trained radiologists and trained emergency room physicians had a high degree of agreement; correlation coefficient 0.9 [0.88–0.93]. In our study, we identified a ‘master Trainer’ who was skilled and experienced with critical care ultrasound. He subsequently trained and supervised the ultrasound measurements obtained by the two investigator critical care physicians between whom there proved to be a high level of agreement as reflected by the high intraclass correlation coefficients.

## Conclusions

In this study of patients admitted to the ICU and with unexplained, non-traumatic coma, we observed that 30 % patients had radiographic cerebral edema. Bedside optic nerve sheath diameter measurement performed well as a rapid, screening test when compared to CT scan for the diagnosis of non-traumatic radiographic cerebral edema. Adding ONSD measurements in the routine evaluation of coma may allow ICU clinicians to rapidly identify patients for whom urgent neuroimaging is indicated.
